# Supplementary material for: Developing Wolbachia-based disease interventions for an extreme environment
Source: PLoS Pathog. 2023 Jan 31;19(1):e1011117. doi: 10.1371/journal.ppat.1011117 (PMC9917306; doi:10.1371/journal.ppat.1011117)
Supplement: S1 Table — (DOCX) [file ppat.1011117.s006.docx]

**Table S1. Nucleotide differences between the *w*AlbB genomes sequenced in this study and the *w*AlbB reference genome.**

| **Position on genome** | **Reference allele** | **Alternate allele** |
| --- | --- | --- |
| 404124 | A | G |
| 507205 | T | C |
| 542038 | G | A |
| 799342 | G | GA |
| 1373220 | C | T |
