# Supplementary material for: Developing Wolbachia-based disease interventions for an extreme environment
Source: PLoS Pathog. 2023 Jan 31;19(1):e1011117. doi: 10.1371/journal.ppat.1011117 (PMC9917306; doi:10.1371/journal.ppat.1011117)
Supplement: S2 Table — (DOCX) [file ppat.1011117.s007.docx]

**Table S2. Primers used in qPCR for detection of DENV2 and *Aedes aegypti* RpS17 nucleic acid**

| **Primer name** | **Sequence 5’- 3’** | **Fragment size (bp)** |
| --- | --- | --- |
| Dengue NS1 F | ACGTGCACACATGGACAGA | 109 bp  (2489bp – 2598bp) |
| Dengue NS1 R | ACTGAGCGGATTCCACAAA |  |
| *Aedes aegypti* RpS17 F | TCCGTGGTATCTCCATCAAGCT | 67bp  (228bp-295bp) |
| *Aedes aegypti* RpS17 R | CACTTCCGGCACGTAGTTGTC |  |
